# Polyunsaturated fatty acids synergize with lipid droplet binding thalidomide analogs to induce oxidative stress in cancer cells

**DOI:** 10.1186/1476-511X-9-56

**Published:** 2010-06-02

**Authors:** László G Puskás, Liliána Z Fehér, Csaba Vizler, Ferhan Ayaydin, Erzsébet Rásó, Eszter Molnár, István Magyary, Iván Kanizsai, Márió Gyuris, Ramóna Madácsi, Gabriella Fábián, Klaudia Farkas, Péter Hegyi, Ferenc Baska, Béla Ózsvári, Klára Kitajka

**Affiliations:** 1 Avidin Biotechnology, Közép fasor 52., Szeged, H-6726, Hungary; 2 Avicor Ltd., Közép fasor 52., Szeged, H-6726, Hungary; 3 Laboratory of Functional Genomics, Institute of Genetics, Biological Research Center, Hungarian Academy of Sciences, Temesvári krt. 62., Szeged, H-6726, Hungary; 4 Institute of Biochemistry, Biological Research Center, Hungarian Academy of Sciences, Temesvári krt. 62., Szeged, H-6726, Hungary; 5 Cellular Imaging Laboratory, Biological Research Center, Hungarian Academy of Sciences, Temesvári krt. 62., Szeged, H-6726, Hungary; 6 National Institute of Oncology, Ráth György u. 7-9., Budapest, H-1011, Hungary; 7 Biotecont Ltd., 1/1. Finn u., Pécs, H-7630, Hungary; 8 First Department of Medicine, University of Szeged, Korányi fasor 8-10., Szeged, H-6720, Hungary; 9 Faculty of Veterinary Science, Szent István University, István u. 2. Budapest, H-1078, Hungary

## Abstract

**Background:**

Cytoplasmic lipid-droplets are common inclusions of eukaryotic cells. Lipid-droplet binding thalidomide analogs (2,6-dialkylphenyl-4/5-amino-substituted-5,6,7-trifluorophthalimides) with potent anticancer activities were synthesized.

**Results:**

Cytotoxicity was detected in different cell lines including melanoma, leukemia, hepatocellular carcinoma, glioblastoma at micromolar concentrations. The synthesized analogs are non-toxic to adult animals up to 1 g/kg but are teratogenic to zebrafish embryos at micromolar concentrations with defects in the developing muscle. Treatment of tumor cells resulted in calcium release from the endoplasmic reticulum (ER), induction of reactive oxygen species (ROS), ER stress and cell death. Antioxidants could partially, while an intracellular calcium chelator almost completely diminish ROS production. Exogenous docosahexaenoic acid or eicosapentaenoic acid induced calcium release and ROS generation, and synergized with the analogs *in vitro*, while oleic acid had no such an effect. Gene expression analysis confirmed the induction of ER stress-mediated apoptosis pathway components, such as GADD153, ATF3, Luman/CREB3 and the ER-associated degradation-related HERPUD1 genes. Tumor suppressors, P53, LATS2 and ING3 were also up-regulated in various cell lines after drug treatment. Amino-phthalimides down-regulated the expression of CCL2, which is implicated in tumor metastasis and angiogenesis.

**Conclusions:**

Because of the anticancer, anti-angiogenic action and the wide range of applicability of the immunomodulatory drugs, including thalidomide analogs, lipid droplet-binding members of this family could represent a new class of agents by affecting ER-membrane integrity and perturbations of ER homeostasis.

## Background

Cytoplasmic lipid-droplets (LDs) are common inclusions of eukaryotic cells. Little is known about the composition or physiological role of LDs, however growing number of evidences imply that LDs are not solely static inclusions for storage of excess lipid, but they are dynamic and functionally active [1-4]. Although LD biogenesis is not well understood, it is assumed that the LDs form within the two leaflets of the ER membrane to function as lipid storage sites [5]. LDs are active inclusions with essential roles in membrane trafficking, cell signaling and distributing specific lipids and proteins [6,7]. LDs are also sites for cytokine storage in inflammatory leukocytes, suggesting that LDs function as inducible intracellular platforms for spatial segregation and organization of signaling leading to inflammatory mediator secretion during inflammation [8]. Under hypoxic conditions or when free fatty acid overload occurs (exogenously from the serum or due to intracellular alterations e.g. when beta-oxidation is blocked) the number of LDs increases [9]. It was shown that LD accumulation occurs *in vivo *at prenecrotic cancer tissues [10] and border zones of experimental myocardial infarcts [11], therefore LDs can be *in vivo *markers of cancer and ischemic insults [12]. When the amount, distribution and the rate of formation of LDs are altered by small molecules, membrane transport and lipid homeostasis could be impaired in cancer cells. Thus, tumor cells are much more dependent upon fatty acid removal than normal cells, where beta-oxidation is not affected by oxygen depletion. Imbalance in lipid homeostasis can finally lead to membrane disruption and activation of lipoapoptosis [13]. As LDs are formed in the ER we hypothesized that specific LD-binding drugs could interfere with LD homeostasis and ER-membrane integrity and could trigger apoptosis through ER stress.

We synthesized novel LD-binding thalidomide analogs (amino-substituted-trifluoro-phthalimides), with strong inherent blue fluorescence, which localize specifically to LDs and at higher concentrations to ER. They possess potent anticancer activities *in vitro *in a variety of cell lines and they, like non-fluorescent immunomodulatory drugs (IMiDs) and thalidomide analogs such as Revlimid (lenalidomide), Actimid and CPS49 [14,15], induce reactive oxygen species (ROS) originating in the ER [16].

Thalidomide is best known as a major teratogen that caused birth defects (stunted limb growth) in up to 12,000 children in the 1960 s. Despite its tragic initial experience, thalidomide has become the subject of major interest because of its wide range of biological activities and newly demonstrated clinical value in infectious disease and cancer [17,18]. Thalidomide and their analogs are currently being used experimentally to treat various cancers and inflammatory diseases (for review see [14,17,18]). Because the use of thalidomide is limited by toxicity and limited efficacy, novel and more potent derivatives are under development [18-21].

Here, we studied the interacting protein targets of amino-substituted-trifluoro-phthalimides and their effects on calcium-release, ROS production, ER-stress and gene expression changes in different treated cancer cells. We also examined whether different fatty acids could influence the effects of our new compounds. Our results might open novel therapeutic strategies by using LD-binding molecules and also emphasize the importance of nutritional lipids on tumor response to anti-neoplastic agents.

## Materials and methods

### Chemicals

2,6-diisopropylphenyl-4/5-amino-substituted-4/5,6,7-trifluorophthalimides (AC-177: 4-cyclopentyl-; AC-202: 5-ethyl-; AC-1041: 4-morpholine-) were synthesized from 2,6-diisopropylphenyl-4-5,6,7-tetrafluorophthalimide [21] and pentylamine, ethylamine or morpholine (Sigma, USA) in chloroform at reflux temperature for 6 h. The 4- (e.g. AC-177 or AC-1041) and 5- (e.g. AC-202) isomers were separated in silica column chromatography by using hexane:chloroform (1:1) eluent. The purity of the compounds was assessed by HPLC. The structure of the compounds was confirmed by NMR or X-ray diffraction (data not shown). Other IMiDs (CPS49: 4,5,6,7-tetrafluoro-2-(2,4-difluorophenyl)isoindol-1,3-dione; TFFI: 4,5,6,7-tetrafluoro-2-(4-fluoro-phenyl)-isoindole-1,3-dione; CPS48: 5-(tetrafluorophthalimido)-pyrimidine-2,4(1 H, 3H)-dione were synthesized according to US 20040077685A1 patent. Lenalidomide (cc5013) was purchased from Hallochem (Chongqing, China), thalidomide and other chemicals from Sigma (St. Louis, MO, USA).

### Intracellular localization

HepG2 and HT168 cells were grown on cover-slips, washed in phosphate-buffered saline (PBS), and fixed for 10 min at room temperature with 4 % p-formaldehyde in PBS supplemented with 5 mM each of MgCl_2 _and EGTA. After washing with PME, LDs were first stained with oil red O for 10 min, than incubated with 10 μM AC compounds in PME for 5 min and mounted in Fluoromount-G mounting solution (Southern Biotechnology Associates, Birmingham, AL). HT168 cells were cultured in glass bottom culture dishes (MatTek Corporation, Ashland, MA). ER was labeled with BODIPY-FL-glibenclamid according to the ER-Tracker Green (Invitrogen, Carlsbad, CA) manufacturer's protocol in live cells. After staining we replaced the solution with fresh Hank's Balanced Salt Solution with calcium and magnesium containing 5 μM AC-202 or AC-1041 and after 5 min the cells were visualized using fluorescence microscope.

Fluorescence microscopy was performed using a confocal microscope (Olympus Fluoview FV1000 Confocal Laser Scanning Microscope) equipped with 20× (N.A 0.75) and 40× oil (N.A 1.3) objectives. We applied a 543 nm laser with Alexa Fluor 546 configuration for detection of oil red O staining of oil bodies and 405 nm laser with DAPI configuration for detection of AC compounds.

*In vivo *staining with AC-202 was performed on SCID mouse after 28 days intra-spleen inoculation of 10^6 ^HT168 cells. 2 h after single oral administration of 200 mg/kg AC-202, mice were sacrificed and livers were frozen and sectioned for confocal microscopy.

### Cell culture studies

HepG2 (hepatocarcinoma) and MCF7 (breast adenocarcinoma), U87 (glioblastoma), HT168 (melanoma), K562, HL60 (myeloid leukemia), U266 (multiple myeloma) cells were obtained from the ATCC collection and maintained in suggested medium in a humidified atmosphere of 95% air and 5% CO_2_. For cytotoxicity assays, 10.000 cells were seeded into one well of 96-well cell culture plates. Effects of the analogs were recorded 24-72 h after treatment. MTS (3-(4,5-dimethylthiazol-2-yl)-5-(3-carboxymethoxy-phenyl)-2-(4-sulfophenyl)-2H-tetrazo-lium) assay was applied to drug treated and control (0.2% DMSO) cells at different concentrations with CellTiter 96^® ^AQueous Assay (Promega, Madison, WI) according to the manufacturer's protocol.

For gene expression analysis cell lines (K562, HL60, U266, HT168, U87, HepG2 and MCF7) (seeded at a density of 5 × 10^4 ^cells/cm^2 ^into 100 mm cell culture dishes) were treated with different drugs at different concentrations (thalidomide: 100 μM, cc5012: 20 μM, CPS48: 20 μM, TFFi: 10 μM, CPS49: 10 μM and AC-1041: 10 μM) for 4 h. Quadruplicate samples for each compound and their respective controls were collected.

### Intracellular calcium ion determination

Cultured K562 cells were attached, using Cell-Tak, to a coverslip (ø 24 mm) forming the base of a perfusion chamber mounted on an Olympus microscope (Olympus, Budapest, Hungary). The K562 cells were bathed in standard Hepes solution at 37°C and loaded with the Ca^2+^-sensitive fluorescent dye FURA 2-AM (4-5 μmol/L) for 30 min. After loading, the cells were continuously perfused with solutions at a rate of 4-5 mL/min. Changes in intracellular Ca^2+ ^concentration ([Ca^2+^]_i_) were measured using a CellR imaging system (Olympus). Each cell was excited with light at wavelengths of 340 nm and 380 nm, and the 380/340 fluorescence emission ratio was measured at 510 nm. One [Ca^2+^]_i _measurement was obtained per second. The standard Hepes-buffered solution contained 130 mM NaCl, 5 mM KCl, 1 mM CaCl_2_, 1 mM MgCl_2_, 10 mM D-glucose and 10 mM Na-Hepes. Cells were loaded with AC-1041 for 2 min.

### ROS determinations

ROS generation was determined by the increase in DCFDA (2',7'-dichlorodihydrofluorescein diacetate, Sigma) fluorescence after drug stimulation. HT168 cells were washed, resuspended in 1% bovine serum albumin in Hanks buffered saline solution (BSA-HBSS) at 10^6 ^cells/mL and maintained at 37°C for analysis. Cells were treated with the indicated drugs (AC-177, CPS49) and with or without different antioxidants (rotenone: 0.1 μM; gluthatione (GSH): 0.4 mM; N-acetyl cysteine (NAC): 0.4 mM; tiron: 0.5 mM; epigallocatechin (EPG): 0.1 mM; buthylsulfoxide (BSO): 0.1 mM; catalase: 100 U; vitamin C: 50 μM), fatty acid (EPA: 50 μM) and BAPTA-AM (50 μM). DCFDA was added 60 min prior to harvest, at 2 μM final concentration. All the other parameters and calculations were done as described before [16].

### Toxicity and teratogenic analysis

Acute toxicology studies were performed on two-months-old male CD/1 mice and Wistar female rats that were kept in a conventional animal house and received conventional food pellets and tap water *ad libitum *throughout the experiments. One dose of AC-202 or AC-1041 at 800 mg/kg or 1.2 g/kg dose was orally given to 10 animals in 0.5% methylhydroxyl-cellulose suspension by gavage (0.45 ml for mice and 2 ml for rats). Additionally, one dose of 2 g/kg of AC-202 was orally given to 10 rats. Toxic effects were recorded for 2 weeks. All animal experiments were performed respecting institutional animal welfare guidelines.

Teratogenic analysis was performed on zebrafish embryos [22]. AC-202, AC-1041 and thalidomide were dissolved in DMSO at final concentration of 1 mM. One in 1000 and 1/200 dilutions were applied into the water. Malformations and death were recorded at 24, 48 and 72 hpf (hours post fertilization).

### Gene expression analysis

Total RNA was purified from drug treated and control (0.2% DMSO) cells at various concentrations with AccuPrep™ RNA purification kit (Bioneer, Daeleon, Korea) according to the manufacturers' protocol, except that DNase I treatment was incorporated.

For DNA-microarray analysis human microarrays of 8-plex format from Agilent Technologies (Palo Alto, CA, USA) with 14,833 probes were used. 1 μg of total RNA from AC-1041-treated and control K562 cells were amplified with the AminoAllyl MessageAmp™ II aRNA Amplification Kit (Ambion, Austin, Texas), and labeled with either Cy5 or Cy3 dyes according to the manufacturer's instructions (Ambion). Each array was scanned at 543 nm (for Cy3 labeling) or at 633 nm (for Cy5 labeling) in Agilent Scanner using the built-in XDR (Extended Dynamic Range) function with 5 μm resolution. Output image analysis and feature extraction was done using Feature Extraction software of Agilent. DNA-microarray study was done in quadruplicates and with dye-swap protocol. The results were deposited at GEO (Gene Expression Omnibus) database with an accession number: GSE14945.

For quantitative real-time PCR (QRT-PCR) total RNA (2 μg) was converted into cDNA with the High-Capacity cDNA Archive Kit (Applied Biosystems, Foster City, CA) and without purification the mixture was applied to QRT-PCR analysis. QRT-PCR was performed on a RotorGene 3000 instrument (Corbett Research, Sydney, Australia) with gene-specific primers http://www.brc.hu/pub/LHD/LHDPrimers.xls and SybrGreen protocol [23]. Curves were analyzed by using dynamic tube and slope correction methods with ignoring data from cycles close to baseline. Relative expression ratios were normalized to the geometric mean of two housekeeping genes, ubiquitin and hypoxanthin phosphoribosyltransferase.

## Results

### Novel thalidomide analogs

**Figure 1:**
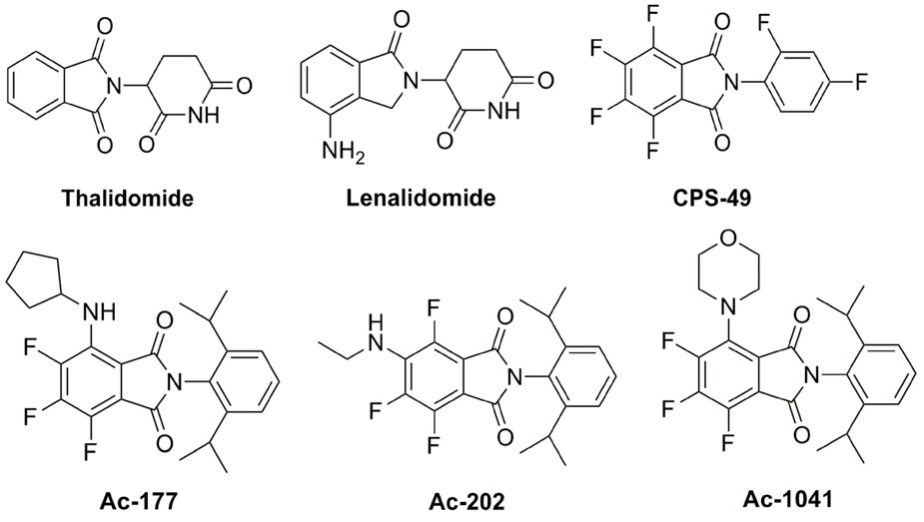
**Chemical structure of thalidomide, lenalidomide, CPS-49, and trifluoro-amino-phthalimides: AC-177, AC-202 and AC-1041**.

Novel thalidomide analogs, 2,6-diisopropylphenyl-4/5-amino-substituted-4/5,6,7-trifluorophthalimides (AC-177: 4-pentyl-; AC-202: 5-ethyl-; AC-1041: 4-morpholine-) were synthesized from 2,6-diisopropylphenyl-4-5,6,7-tetrafluorophthalimide [24] (see structures in Fig. 1). Several primary and secondary amine compounds including heterocyclic structures were chemically reacted and novel thalidomide derivatives were prepared and characterized (unpublished results). All possessed a strong blue fluorescence (e.g.: AC-202: Λ_ex_: 377 nm, Λ_em_: 481 nm), that made cell/animal analyses more convenient.

### Intracellular localization

**Figure 2:**
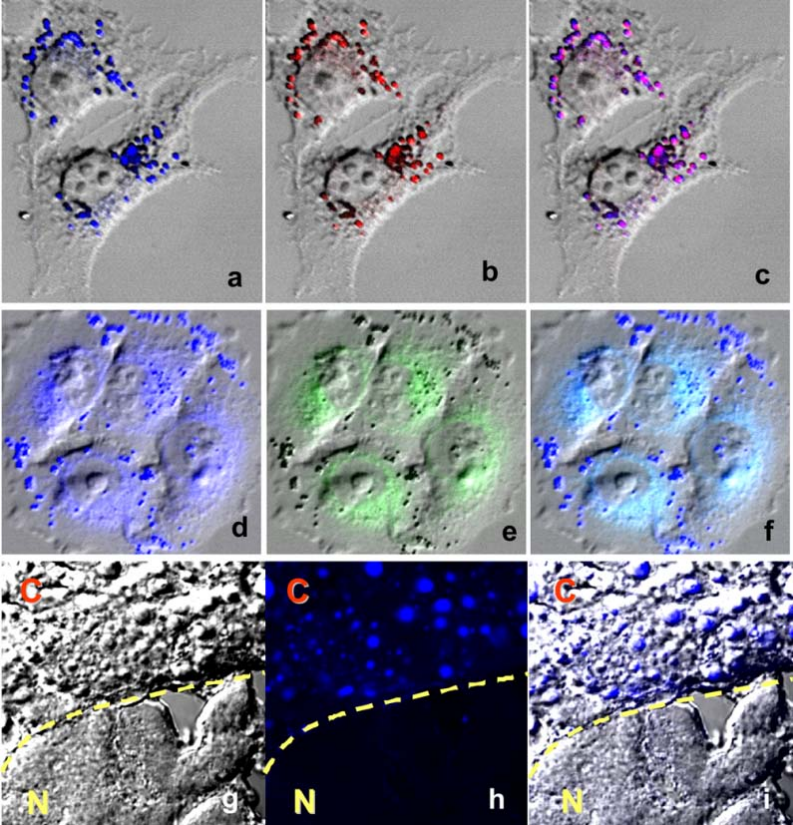
**Intracellular localization of AC-202**. HT168 human melanoma cells were double-stained with the blue fluorescent AC-202 (a. 5 μM; d. 20 μM), lipid droplet-specific dye (oil red O) (b), and endoplasmic reticulum-specific dye (ER-Tracker™ Green) (e). Purple color derives from co-localization of oil red O and AC-202 (c). Cyan color derives from co-localization of ER-Tracker™ and AC-202 (f). Pictures d-f were recorded with higher laser power. In vivo staining of lipid droplets in prenecrotic cancer tissues (HT168 xenograft) in liver of SCID mouse by AC-202 (g-i). "C" denotes for cancer tissue, "N" for normal. Cancer and normal cells are separated by a dashed line.

Fluorescence confocal microscopic study was undertaken to determine the subcellular localization of the new thalidomide analogs in human cells. AC compounds exhibited specific staining of LDs at 5 μM concentration assessed by complete co-localization with oil red O, an LD-specific dye in HT168 human melanoma cells (for AC-202 see Fig. 2a-c). When higher concentrations and higher laser power were applied, AC-202 also showed ER localization (Fig. 2d-f). Complete LD co-localization for all AC compounds listed in the Materials and Methods section were reproducible on other cell lines (MCF7, HepG2, A549, K562, HL60, U87, U266), as well (data not shown).

*In vivo *staining of pre-necrotic, necrotic tumor tissues (human HT168 melanoma cells) in SCID mouse liver could be seen after single oral administration of 200 mg/kg AC-202 (Fig. 2g-i). Lipid droplets were accumulated in tumor tissues and were stained by the inherent fluorescence of the analog AC-202, while normal liver tissues lack LDs and therefore, had much less fluorescent signals.

### In vitro effects on cancer cells

**Figure 3:**
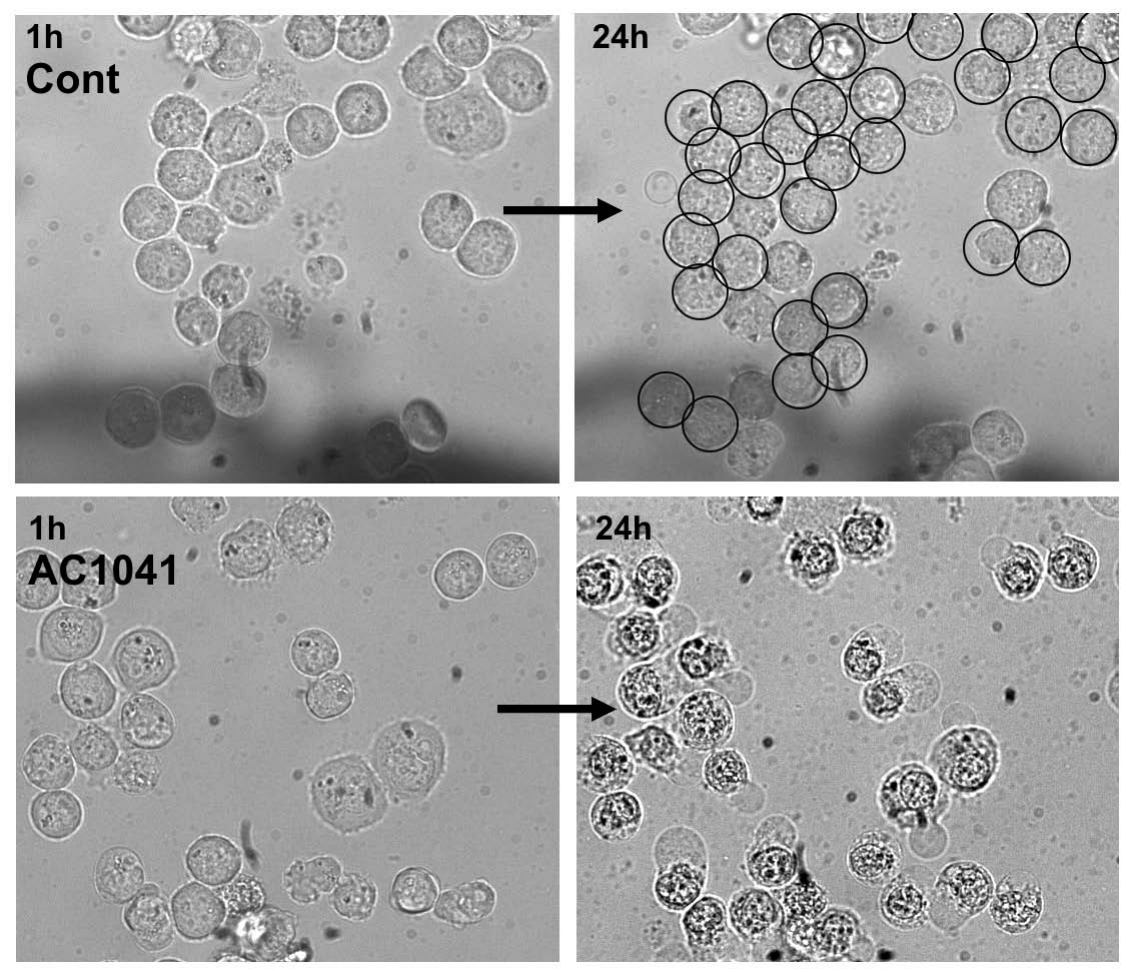
**Cytotoxic activity of 10 μM AC-1041 on human erythroleukemia cells**. Pictures were recorded from the same area after 1 h and 24 h treatment. Cont: control cell lines treated with vehicle (0.1% DMSO). Cell divisions between 4 h-24 h are labeled with circles.

Amino-trifluoro-phthalimides exerted potent anticancer activities *in vitro*. Cytotoxicity was evident after 4-16 h exposure depending on the cell type (data not shown). In human erythroleukemia cells (K562), which showed significant resistance against several thalidomide analogs [15], amino-trifluoro-phthalimides were effective below 10 μM concentration (for AC-1041 see Fig. 3).

Cytotoxicity could be observed in all cell lines analyzed: in leukemia (K562, HL60), myeloma (U266), glioblastoma (U87), melanoma (HT168), breast cancer (MCF7) and hepatocellular carcinoma (HepG2). The most effective compound was AC-1041 with IC_50 _values of 5-15 μM. The most sensitive cell lines were the leukemia cells (5 μM), while melanoma, hepatocellular carcinoma and glioblastoma cell lines were the most resistant (15 μM).

### Elevation of intracellular calcium ion and ROS

**Figure 4:**
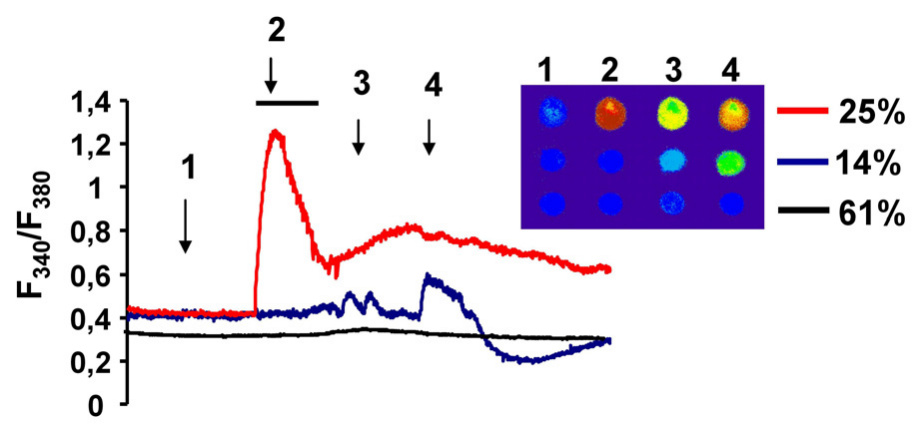
**Representative experimental traces showing the different effects of AC-1041 on [Ca^2+^]_i _in perfused K562 cells**. Shown are the typical patterns of [Ca^2+^]_i _changes in the cells perfused with AC-1041. Increase in [Ca^2+^]_i _is denoted by a change from a ''cold'' color (blue) to a ''warmer'' color (yellow to red). Pictures 1-4 were taken at the times indicated in the graph.

In human erythroleukemia cells (K562) LD-binding amino-trifluoro-phthalimides induced intracellular calcium ion release from the ER (see Fig. 4 for induction by AC-1041 at 10 μM concentration) as detected by measurement of the Ca^2+^-sensitive fluorescent dye FURA 2-AM for 30 min after drug administration. AC-1041 evoked a large initial peak in [Ca^2+^]_i _followed by a sustained plateau in 25% of the cells, however, in 14% of the cells AC-1041 evoked repetitive [Ca^2+^]_i _transients. 61% of the cells did not (or very slightly) respond.

**Figure 5:**
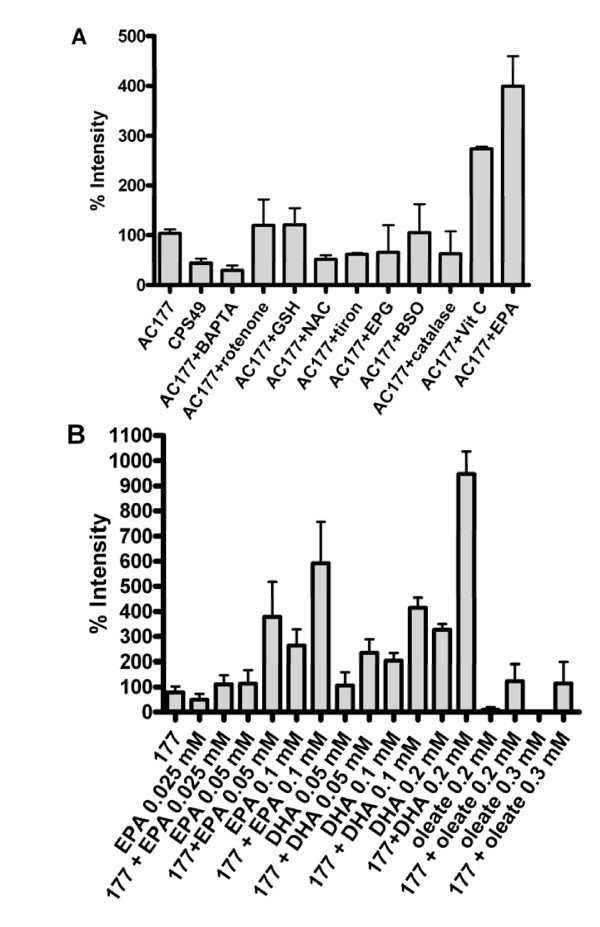
**Effect of different antioxidants and EPA on AC-177-induced oxidative stress in HT168 human melanoma cells (A)**. Stimulated increase in ROS formation after treatment of HT168cells with AC177 (5 μM) alone or in the presence of different concentrations of EPA, DHA and oleate (B).

To investigate whether amino-trifluoro-phthalimides exert a pro-oxidative effect, the presence of ROS in cancer cells was measured after exposure of the cells to the analogs. AC-177 and other analogs generated significant levels of ROS in HT168 cells, while control compounds cc5013, thalidomide and CPS49 showed only minimal elevation compared with that of untreated HT168 cells (for CPS49 and AC-177 ROS levels, see Fig. 5A). The effects of different antioxidants (rotenone, GSH, NAC, tiron, EPG, BSO, catalase and vitamin C) and polyunsaturated fatty acid EPA were also recorded on ROS production (Fig. 5A). Among the antioxidants only NAC and tiron could diminish ROS production. Interestingly, vitamin C and EPA raised the AC-177-induced ROS levels. Moreover, the addition of different polyunsaturated fatty acids (PUFAs) (EPA or DHA) in combination with AC-177 resulted in synergistic effects on ROS production (Fig. 5B). When oleic acid was used no such an effect could be detected. The synergistic effect of EPA or DHA was concentration dependent. This could be seen with the LD-binding amino-trifluoro-phthalimides treatment, but the effect was lacking or was more moderate for other thalidomide analogs (for CPS49 see Fig. 5A).

To reveal the correlation of induction of intracellular calcium ion and ROS production of the analogs we studied the effects of cell-permeable calcium chelating agent, BAPTA-AM together with AC-177 and with PUFAs. We found that BAPTA-AM almost completely abolished ROS formation of AC-177 alone or with the PUFAs used (Fig 5A).

### Toxicity and teratogenicity

Acute toxicology studies were performed on CD/1 mice and Wistar rats. AC-202 or AC-1041 was administered orally. There were no toxic effects up-to very high concentrations (LD50: 1 g/kg for mouse and 1.2 g/kg for rat) for adult animals, but caused severe diarrhea and appendicitis and finally death (80% mortality) in rats after 7-10 days at higher doses (2 g/kg).

**Figure 6:**
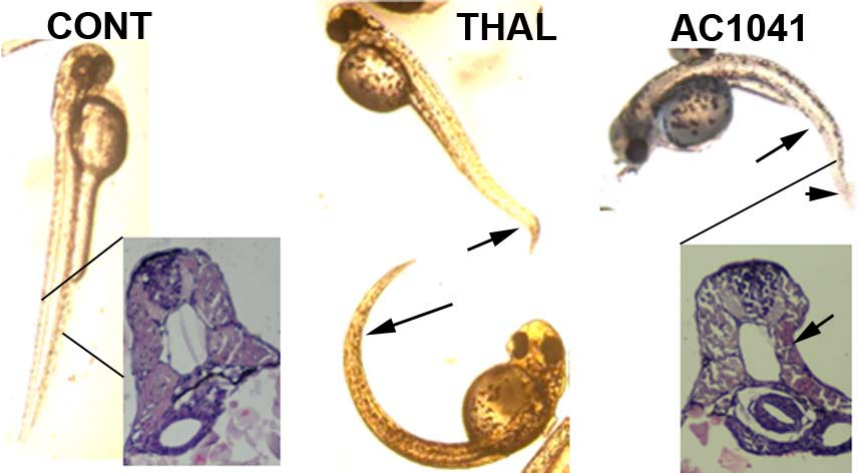
**Teratogenic activity of AC-1041 on zebrafish embryos**. Control embryo (CONT), Thalidomide treated (5 μM; THAL) and AC-1041 treated (5 μM; AC-1041) embryos (b), photo was taken at 3 dpf (days post fertilization). Histological sections show differences in muscle structure after treatment. Arrows indicates malformations.

AC compounds exhibited strong teratogenic effects on zebrafish embryos at low (micromolar) concentrations as can be seen on Fig. 6. The tail of the fish is bended and distorted (shortened and having an "S"-shaped break at the end) when compared to control animals. The same distortion effects can be seen for thalidomide treated animals. At the distortion site defect in muscle formation can be seen (in Fig. 6 arrows show distortion sites of the treated animals).

### Gene-expression changes of treated cancer cells

To further investigate the mechanisms underlying growth inhibitory effects of amino-trifluoro-phthalimides on cancer cells, different cell lines (K562, HL60, U266, HT168, U87, HepG2 and MCF7) were treated with AC-1041 at an IC_50 _concentration for 4 h. Using treated K562 cells a gene array analysis was performed with DNA-microarrays. Microarray data were deposited at GEO (Gene Expression Omnibus) database with an accession number GSE14945. AC-1041 activated expression of a set of genes involved in the ER stress (*ATF3, DDIT3 (GADD153), HERPUD1, PP1R15A, DNAJB1, DNAJB4, DNAJB9, FOS, FOSB, JUN, NR4A1, NR4A2, RELB, TRIB3, LCRF*). Other set of induced genes are coding for tumor suppressor proteins (*TP53, ING3, LATS2*) or part of the JNK/p38 pathway (*DUSP1, DUSP8, DUSP10*). A specific marker gene of cell division, *CCND1 *coding for cyclin D1 was down-regulated. Significant repression of *CCL2 *(chemokine C-C motif ligand 2, or MCP-1), and induction of *SNX16 *(sorting nexin 16) could be detected. To confirm the expression of these genes QRT-PCR was done (K562 column in Table 1). In order to assess the effects of AC-1041 on other cell lines (HL60, U266, HT168, U87, HepG2 and MCF7) the expression of these genes were also studied (Table 1). While almost in all cell lines the induction of ER stress-related genes could be observed, changes at the mRNA level of *TP53, ING3*, the *DUSP *family and *SNX16 *could be recorded only in the sensitive cells (leukemia and myeloma) and not in other cell lines.

**Table 1:** Expression changes in different cells after AC-1041 treatment.

Code	Acc. No.	Gene product	K562	HL60	U266	HT168	U87	HEPG2	MCF7
CCL2	NM_002982	Chemokine (C-C motif) ligand 2	**0.31**	**0.35**	**0.37**	n.d.	0.80	**0.34**	**0.51**
ATF3	NM_004024	Activating transcription factor 3	***10.21***	***2.75***	***8.17***	***18.29***	***4.10***	0.66	***4.32***
DDIT3	NM_004083	DNA-damage-inducible transcript 3	***3.73***	***3.28***	***6.19***	***3.67***	***2.84***	***2.91***	***6.43***
LCRF	NM_153607	Luman/CREB3 rercruitment factor	***4.19***	***10.02***	***2.02***	***12.49***	***2.38***	***2.45***	n.d.
DNAJB1	NM_006145	DnaJ (Hsp40) homolog B1	***10.14***	***3.23***	***4.15***	***10.41***	***5.93***	***5.67***	***2.09***
HERPUD1	NM_014685	ER stress-inducible, ubiquitin-like 1	***2.76***	1.76	1.02	***7.63***	***2.57***	***3.81***	***2.03***
HSPH1	NM_006644	Heat shock 105kDa/110kDa protein 1	***3.64***	***2.69***	1.70	***3.26***	***1.88***	***2.27***	0.89
TP53	NM_000546	Tumor protein p53	***2.37***	***7.62***	1.07	1.31	1.01	1.05	***4.02***
ING3	NM_019071	Inhibitor of growth family, member 3	1.63	***2.10***	***1.80***	***1.92***	1.59	0.72	***2.51***
LATS2	NM_014572	LATS, large tumor suppressor, homolog 2	***2.62***	1.73	1.20	***3.57***	***2.11***	**0.24**	0.65
CCND1	NM_053056	Cyclin D1	**0.46**	1.13	1.00	0.85	1.19	***2.12***	1.12
DUSP1	NM_004417	Dual specificity phosphatase 1	***3.77***	1.30	***5.29***	0.94	1.66	0.94	1.62
DUSP8	NM_004420	Dual specificity phosphatase 8	***9.23***	***5.81***	***7.94***	1.01	***2.68***	***1.91***	***1.80***
DUSP10	NM_007207	Dual specificity phosphatase 10	***2.86***	***2.22***	***2.05***	0.81	1.30	1.21	***3.35***
SNX16	NM_022133	Sorting nexin 16	***3.19***	***3.07***	1.39	1.07	0.75	1.24	***1.92***

Down-regulated gene expression values (<1.80-fold) are indicated by bold, underlined numbers, induced expression values (>1.80-fold) by bold and italics.

## Discussion

The potential to alter tumor-lipid homeostasis through nutritional intervention was established from observations that tumors derive their fatty acids from the *de novo *synthesis pathway that generates stearic and oleic acid [25]. When the synthesis is over-balanced or when fatty acid degradation by beta-oxidation is repressed due to hypoxic conditions, cells direct their excess fatty acids into intracellular lipid depots, into LDs. The lipid in these droplets undergoes turnover via hydrolysis and re-synthesis providing excellent target for affecting tumor cell survival. Moreover LDs have recently been suggested to represent target compartments for fatty acid scavenging to protect cells from lipoapoptosis [26]. We hypothesized that small molecules interacting with LDs cause imbalance in lipid homeostasis resulting in membrane disruption that can finally lead to apoptosis.

We synthesized novel thalidomide analogs (2,6-dialkylphenyl-4/5-amino-substituted-5,6,7-trifluorophthalimides) that possess bright blue fluorescence. They exerted potent anticancer activities *in vitro *in different cell lines: leukemia (K562, HL60), myeloma (U266), glioblastoma (U87), melanoma (HT168), breast cancer (MCF7) and hepatocellular carcinoma (HepG2). In human erythroleukemia cells (K562), which showed significant resistance against several thalidomide analogs [15], amino-trifluoro-phthalimides were effective below 10 μM concentration. We showed that amino-trifluoro-phthalimides localize to LDs and at higher concentrations to ER. Moreover, oral administration of AC-202 resulted in staining LD of metastatic melanoma grown in the liver of xenograft mouse (Fig. 2g-i), suggesting not only good oral bioavailability but also accumulation of the drugs in LDs of pre-necrotic cancer tissues.

Based on our hypothesis regarding to ER membrane disturbance of the LD-binding compounds and previous findings on "redox-reactive" thalidomide analogs [15], we studied the effects of the amino-trifluoro-phthalimides on intracellular calcium release and ROS formation. In K562 cells they induced intracellular calcium ion release from the ER. They evoked a large initial peak in [Ca^2+^]_i _followed by a sustained plateau or induced repetitive [Ca^2+^]_i _transients (Fig. 4.). AC-177 and other analogs generated significant levels of ROS in HT168 (and K562, not shown) cells, while control compounds cc5013, thalidomide and CPS-49 showed only minimal elevation compared with that of untreated cells. ROS formation could be substantially decreased by the cell-permeable calcium chelator, BAPTA-AM, which suggests correlation between the elevation of intracellular calcium ion concentration and ROS production induced by the analogs. Different antioxidants partially diminished ROS production, but significant reduction could be achieved only with NAC and tiron. Interestingly, vitamin C elevated the AC-177 induced ROS formation. The same effect could be observed when DHA or EPA was added in combination with AC-177 (Fig. 5B). This effect could be explained by affecting the membrane structure of the ER by polyunsaturated fatty acids and sensitization of cells against ROS forming agents. However, oleic acid does not display such effect neither alone nor in combination with AC-177.

A species-specific conversion to free radical intermediates in embryonic tissue was suspected to be the main reason for thalidomide teratogenicity [27]. Therefore, we studied the teratogenic effects of the LD-binding thalidomide analogs and thalidomide as a control on zebrafish embryos. AC-177, AC-202 and AC-1041 caused severe morphological changes in the fish like distortion of muscle development and curves in their tails at micromolar concentrations (Fig. 6). The same pathological phenomena could be detected in the case of thalidomide and other thalidomide analogs. It is interesting that zebrafish is sensitive to thalidomide, whereas mice and rats are not. Similar sensitivity for aflatoxins and dimethylnitrosamine could be found in rainbow trout to generate primary liver carcinoma [28,29]. Besides the teratogenicity of the analogs, in initial animal studies, high levels of AC-202 and AC-1041 were well tolerated in adults, and they showed no toxicity even at 1 g/kg dose. Therefore, the LD-binding thalidomide analogs are ideal candidates for further in vivo studies and possibly for entering into clinical trials against cancer.

Thalidomide and its analogs have been discovered to have various biological activities, such as anti-inflammatory, anti-angiogenic, cyclooxygenase (COX)-inhibitory and microtubule-perturbing activities [24,30-32]. However, there are only limited numbers of studies revealing the mechanisms of action of different analogs by using gene expression fingerprinting or clustering of different biological patterns. In the present study we applied DNA-microarrays and QRT-PCR to identify early genes affected by the analogs. AC-1041 induced the expression of 251 genes and repressed 37 genes (data for all of the 14,833 genes examined can be seen at GEO database with an accession number of GSE14945) in K562 cells after 4 h treatment. Microarray results showed that a large number of genes associated with the ER stress response are rapidly induced by AC-1041. This suggests that AC-1041-induced apoptosis is coupled to the ER stress response. During this response, several pro-survival and pro-apoptotic signals are activated and, depending on the extent of the ER stress, cells survive or when functions of the ER are severely impaired, they undergo apoptosis [33,34]. One of the most universal components of the ER stress-mediated apoptosis pathway is *C/EBP *homologous protein (*CHOP*), also known as *GADD153 *or *DDIT3 *[35], which is markedly induced by AC-1041. Other induced genes code for proteins that are part of the JNK/p38 pathway (*DUSP1, DUSP8, DUSP10*) and have roles in regulation of cellular stress responses [36]. Tumor suppressor genes (*TP53, ING3, LATS2*) were also induced, while a specific marker gene of cell division, *CCND1 *coding for cyclin D1 was down-regulated. Interestingly, expression of other genes that could explain multiple action of thalidomide analogs *in vivo *were also changed. *CCL2 *was repressed and *SNX16 *was induced by more than 3-fold as assessed by QRT-PCR. CCL2 is a member of the cytokine/chemokine superfamily and is known to promote the migration of monocytes and macrophages to sites of inflammation and having roles in the tumorigenesis and metastasis of several solid tumors [37,38]. It was also demonstrated that CCL2 acts as a direct mediator of angiogenesis [39]. SNX16 directs the sorting of EGFR from early endosomes to lysosomes and thus has critical role in the termination of EGF-induced cell signaling [40,41].

To further investigate the mechanisms underlying growth inhibitory effects of amino-trifluoro-phthalimides different cell lines (K562, HL60, U266, HT168, U87, HepG2 and MCF7) were treated with AC-1041 at an IC_50 _concentration and gene expression changes were followed for 15 key genes (Table 1). The induction of ER stress-related genes and repression of CCL2 could be observed in all cell lines, suggesting common mechanism of action. Differences could be seen in case of *TP53, ING3, DUSP *family and cyclin D1, which correlated with the chemo-sensitivity of the cells.

Considering the positive effects of AC-1041 on altering *CCL2 *and *SNX16 *expression, it is likely that these effects (angiogenesis and tumorigenesis inhibition by *CCL2 *repression, and epidermal growth factor receptor down-regulation by *SNX16 *induction) in addition to induction of ER stress pathways are important and specific for the biological effects of amino-trifluoro-phthalimides on cancer cells.

## Conclusions

Here we report, that interference with lipid homeostasis and ER membrane integrity of LD-binding thalidomide analogs and the subsequently induced ER stress are crucial steps in the cytotoxic action of these molecules. Additionally to this common pathway that are affected by "redox-reactive" thalidomide analogs, repression of *CCL2 *and induction of *SNX16 *could explain the multiple therapeutic actions of IMiDs. Our results might open novel therapeutic strategies for anti-cancer drug discovery and therapy by using LD-binding molecules and also emphasize the importance of anti-neoplastic agents that affect vesicle transport and the homeostasis of the ER.

## Abbreviations

TAG: triacylglycerol; LD: lipid droplet; ER: endoplasmic reticulum; EPA: eicosapentaenoic acid (20:5: n-3); QRT-PCR: quantitative real-time PCR; MTS: 3-(4,5-dimethylthiazol-2-yl)-5-(3-carboxymethoxyphenyl)-2-(4-sulfophenyl)-2H-tetrazolium; ROS: reactive oxygen species; GSH: gluthatione; NAC: N-acetyl cysteine; BSO: buthylsulfoxide;

## Competing interests

PGL, CEO of Avidin Ltd., has interests in the commercialization of novel thalidomide analogs studied in the current article. Avidin Ltd. also has patent in the area of thalidomide analogs.

## Authors' contributions

Fluorescent thalidomide analogs were synthesized by IK, MG, RM. Confocal laser scanning microscopy was performed by FA, LZF. Affinity chromatography, polyacrylamide gel-electrophoresis and mass spectrometry involved LZF, GF, EM. Cytotoxicity assays involved BO, LZF. Intracellular Ca^2+ ^measurements were performed by PH, KF, GF. GF also participated in ROS generation which was determined by flow cytometry. Gene expression analysis involved LZF, LGP. IM and FB was performed teratogenic analysis. Data analysis, drafting of the manuscript was performed by LGP, KK, CV and ER. All authors read and approved the final version.
